# An Improved *Agrobacterium*-Mediated Transformation and Genome-Editing Method for Maize Inbred B104 Using a Ternary Vector System and Immature Embryos

**DOI:** 10.3389/fpls.2022.860971

**Published:** 2022-05-04

**Authors:** Minjeong Kang, Keunsub Lee, Todd Finley, Hal Chappell, Veena Veena, Kan Wang

**Affiliations:** ^1^Department of Agronomy, Iowa State University, Ames, IA, United States; ^2^Crop Bioengineering Center, Iowa State University, Ames, IA, United States; ^3^Interdepartmental Plant Biology Major, Iowa State University, Ames, IA, United States; ^4^Plant Transformation Facility, Donald Danforth Plant Science Center, St. Louis, MO, United States

**Keywords:** bialaphos, CRISPR-Cas9, helper plasmid, tissue culture, *Zea mays*

## Abstract

For maize genome-editing and bioengineering, genetic transformation of inbred genotypes is most desired due to the uniformity of genetic background in their progenies. However, most maize inbred lines are recalcitrant to tissue culture and transformation. A public, transformable maize inbred B104 has been widely used for genome editing in recent years. This is primarily due to its high degree of genetic similarity shared with B73, an inbred of the reference genome and parent of many breeding populations. Conventional B104 maize transformation protocol requires 16–22 weeks to produce rooted transgenic plants with an average of 4% transformation frequency (number of T0 plants per 100 infected embryos). In this Method paper, we describe an advanced B104 transformation protocol that requires only 7–10 weeks to generate transgenic plants with an average of 6.4% transformation frequency. Over 66% of transgenic plants carried CRISPR/Cas9-induced indel mutations on the target gene, demonstrating that this protocol can be used for genome editing applications. Following the detailed and stepwise procedure described here, this quick and simplified method using the *Agrobacterium* ternary vector system consisting of a T-DNA binary vector and a compatible helper plasmid can be readily transferable to interested researchers.

## Introduction

Maize is the most produced grain crop for humans and livestock throughout the world. It is grown in more countries than any other crop and serves as an important model plant system for fundamental studies (Freeling and Walbot, 1994). Recent advances in genomics and molecular biology tools have enabled rapid identification and isolation of plant genes on a large scale. Especially, the RNA-guided endonucleases adopted from the prokaryotic immune system, i.e., Clustered Regularly Interspaced Short Palindromic Repeat (CRISPR) and CRISPR-associated protein (Cas) systems, have been widely used for precise gene editing in plants and agricultural research (Zhu et al., 2020). CRISPR-Cas9 represents one of the most efficient gene editing tools and has a great potential for gene functional analysis (Zhu et al., 2020). However, determining the functions of thousands of genes and applying that knowledge to crop improvement is now one of the major challenges facing plant biologists. Plant genetic transformation is a key technology for functional analysis of genes *via* strategies including complementation, overexpression, gene silencing, or genome editing, therefore, it is critical to improve the plant genetic transformation protocols to take full advantage of the revolutionary genome engineering tools.

Maize transformation can be achieved by either biolistic- or *Agrobacterium*-mediated methods (Kausch et al., 2021). While private laboratories are now routinely operating high throughput transformation pipelines, many public research groups face challenges in obtaining transgenic and genome-edited maize plants efficiently, especially when conducting transformation using maize inbred genotypes.

Like many other plant transformation processes, maize transformation is genotype-dependent (Que et al., 2014). The early maize transformation successes used specific hybrid maize genotypes such as A188 × B73 (Gordon-Kamm et al., 1990) and Hi Type II (Hi II, Frame et al., 2002) and a few old inbreds such as A188 (Ishida et al., 1996). To properly understand gene functions and compare changes made on the genes, a transgenic analysis should be performed in an inbred background. Frame et al. (2006) conducted experiments on ten maize inbred lines in an attempt to identify inbreds that can be transformed. Three inbred genotypes, B104, B114, and Ky21, were found to be transformable using the *Agrobacterium*-mediated infection of immature embryos (Frame et al., 2006). Among the three inbreds, B104 is particularly of interest because it was derived from the same populations as inbred B73 (Hallauer et al., 1997). B73 is a public inbred that currently serves as the reference genome (Schnable et al., 2009). B104 shares the same genetic background as B73; both were derived from the Iowa Stiff Stalk Synthetic lines10 (Hallauer et al., 1997). They share 93% similarity as calculated by the TYPSimSelector tool available at MaizeGDB (Romay et al., 2013; Manchanda et al., 2016).

Transformation of B104 uses Murashige and Skoog (MS) basal salts for callus initiation and regeneration. Interestingly, the same media regime and treatments did not work for B73 transformation (Frame et al., 2006). Because of the difficulty in B73 transformation, B104 has been used widely in the maize research community as an alternative inbred for functional gene analysis in recent years (Char et al., 2017; Sun et al., 2017; Lee et al., 2019). B104 can be transformed using both *Agrobacterium* and biolistic methods (Raji et al., 2018) with varied frequencies. Here, transformation frequency is defined as the number of herbicide (bialaphos) resistant events per 100 infected immature embryos. The average frequency of the *Agrobacterium*-mediated method was 4%, while the frequency of the biolistic approach was 6–13% (Raji et al., 2018).

In general, the process of B104 transformation takes longer than that of Hi II transformation. Counting from the day of infection/bombardment to the day of moving regenerated plants to the soil, B104 transformation takes about 160 days (Frame et al., 2006; Raji et al., 2018), whereas Hi II transformation takes about 110 days (Frame et al., 2015). Because B104 produces type I callus from immature embryos, it can be challenging in managing effective regeneration of transformed cells, especially for less experienced researchers.

To overcome the genotype dependency in monocots transformation, researchers have been exploring plant genes that can promote somatic embryogenesis (Lardon and Geelen, 2020). Lowe et al. (2016) showed that when they introduced morphogenic genes *Baby boom* (*Bbm*) and *Wuschel2* (*Wus2*) into the cells of transformation-recalcitrant grass species, they could produce transgenic plants in these genotypes that were normally not amenable for genetic transformation. Subsequently, it was also shown that maize B73 inbred could also be transformed using the vectors containing the morphogenic genes (Mookkan et al., 2017; Masters et al., 2020). Importantly, the protocol developed by Lowe et al. (2016) allowed a significant reduction of time required for transformation. This morphogenic genes-enabled protocol was termed as “QuickCorn” method (Masters et al., 2020).

While the morphogenic genes can stimulate embryogenesis, they also regulate and impact other genes and their expressions in plant cells (Ikeuchi et al., 2019). The persistent presence of extra morphogenic genes in plant cells can negatively affect plant development and maturity (Boutilier et al., 2002; Zuo et al., 2002). To overcome this issue, all transformation vectors carrying the morphogenic genes also contain a *cre-loxP* recombinase system that can remove the morphogenic genes from the transgene after the initial transformation stage (Lowe et al., 2016). However, the presence of both morphogenic gene cassettes (∼7 kb) and the *cre-loxP* system (∼3.5 kb) substantially increases the T-DNA size on the transformation vectors (Lowe et al., 2016) and makes it difficult for further sequence modifications in the vectors. Moreover, larger T-DNA size on a transformation vector often leads to lower transformation frequencies (Park et al., 2000).

Here, we describe an improved protocol for B104 transformation using the *Agrobacterium*-mediated method. Like the original B104 transformation protocol (Frame et al., 2006), this protocol uses maize immature embryos harvested 10–12 days post-pollination. We use an *Agrobacterium* strain harboring a ternary vector system: a conventional T-DNA binary vector and a compatible helper plasmid that carries additional copies of *Agrobacterium* virulent (*vir*) genes. Extra copies of *vir* genes have been shown to greatly enhance maize transformation frequencies (Ishida et al., 1996; Anand et al., 2018). One major difference in this protocol is the adoption of the media regime from the QuickCorn method (Hoerster et al., 2020; Masters et al., 2020). These changes improve tissue responsiveness and overall regenerability. Most importantly, it greatly reduces the duration of the transformation process from 160 to 60 days, counting from the day of infection to the day of moving regenerated plants to soil. By delivering a CRPSPR/Cas9 system *via* the T-DNA, we demonstrate that this improved B104 transformation protocol can be used for efficient genome editing applications.

## Materials and Equipment

### Plant Material, Constructs, and *Agrobacterium* Strain

**FIGURE 1:**
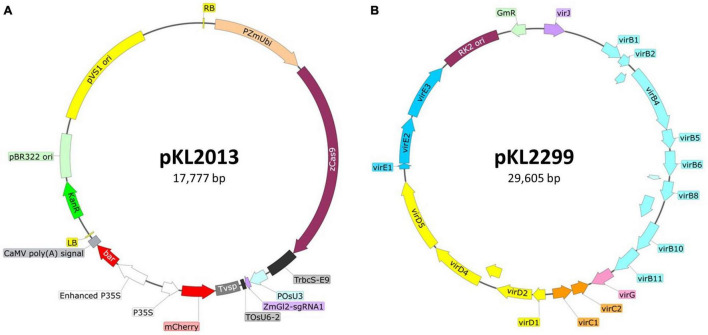
Schematic illustration of the ternary vector system used for maize B104 transformation. **(A)** Binary construct pKL2013 (McCaw et al., 2021), 17,777 bp. PZmUbi, maize polyubiquitin gene promoter; zCas9, maize codon-optimized *Cas9* from *Streptococcus pyogenes*; TrbcS-E9, transcriptional terminator from *Pisum sativum rbcS*-E9 gene; POsU3, a promoter from *Oryza sativa* U3 small nucleolar RNA (snoRNA) gene; ZmGl2-sgRNA1, single-guide RNA targeting maize *glossy2* gene (Lee et al., 2019); TOsU6-2, terminator from *O. sativa* OsU6-2 snoRNA gene; Tvsp, terminator from soybean vegetative storage protein gene; mCherry, red fluorescent protein; P35S, CaMV 35S promoter; bar, bialaphos resistance gene; RB, the T-DNA right border repeat; LB, the T-DNA left border repeat; KanR, kanamycin resistance gene cassette; pBR322 ori, high copy number origin of replication for *E. coli*; pVS1 ori, the origin of replication from the plasmid pVS1. **(B)** Ternary construct or helper plasmid pKL2299 (this work), 29,605 bp. *virB1-virJ*, virulence genes from the *Agrobacterium tumefaciens* Bo542 tumor-inducing plasmid (pTiBo542); RK2 ori, the origin of replication from the broad host range RK2 plasmid; GmR, gentamicin resistance gene cassette.

• Maize inbred B104: Seed can be obtained from the USDA Agricultural Research Service seed repository.^1^
• Plant material: Greenhouse-grown B104 embryo donor ears are harvested 10–12 days after pollination (depending upon the weather and day length) when immature zygotic embryos are 1.6–2.0 mm long. After harvest, maize ears (in their husks and inside their pollination bag) can be stored in a laboratory refrigerator (4°C) for 1–3 days prior to use.
• Binary vector pKL2013 (McCaw et al., 2021; Figure 1A): The T-DNA carries (1) 2X CaMV 35S promoter (P35S) driving the bialaphos resistant (*bar*) gene as a selectable marker, (2) a single P35S driving mCherry gene as a visual marker; (3) maize ubiquitin promoter driving a maize-codon-optimized *Cas9*, and (4) rice U3 promoter driving a guide RNA (gRNA) targeting maize *glossy2* (*Gl2*) gene (McCaw et al., 2021).
• Ternary helper plasmid pKL2299 (this work; Figure 1B): It carries the majority suite of *Agrobacterium* virulence genes from Ti plasmid pTiBo542 (Komari et al., 1986). The additional copies of *vir* genes have been shown to markedly enhance maize transformation frequencies when using *Agrobacterium* strain LBA4404 (Anand et al., 2018). To ensure vector compatibility, pKL2299 has the RK2 origin of replication. Therefore, this plasmid can be used as a ternary vector pairing with any binary vector plasmid that has a pVS1 origin of replication.
• *Agrobacterium tumefaciens* strain LBA4404Thy-: This is an auxotrophic version of LBA4404 with the thymidylate synthase gene (*thyA*) deleted from the chromosome (Ranch et al., 2012; Anand et al., 2018).
• *Agrobacterium* strain carrying the constructs: LBA4404Thy- harboring both pKL2013 and pKL2299 is called KL2013vir/thy and used in this study.

### Plant Growth Supplies

• Metromix 360 (Sungro, Agawam, MA, United States).
• Pro-Mix BRK20 (Pro-Mix, Quakertown, PA, United States).
• 1801 deep inserts (T.O. Plastic, Clearwater, MN, United States).
• Standard Flat with drain holes, STF-1020-OPEN (T.O. Plastic, Clearwater, MN, United States).
• Standard 2.5-gallon (9.4 L) nursery pots (Nursery Supplies Inc., Chambersburg, PA, United States).
• Humi-dome (Hummert International, Earth City, MO, United States).
• Tomato maker 4-2-6 (Organic labs, Fort Pierce, FL, United States).
• Osmocote 15-9-12 (Everris, Geldermalsen, Netherlands).
• Sprint 330 Iron chelate (BASF, Ludwigshafen, Germany).
• Pollination apron.
• Scissors.
• 70% Ethanol to sterilize scissors.
• Shoot bags (Lawson Pollinating Bags, Northfield, IL, United States).
• Tassel bags (Lawson Pollinating Bags, Northfield, IL, United States).
• Permanent marker–Chisel tip for broad strokes, black, standard (NOT ethanol resistant) recommended to avoid fading in the sun.
• Stapler and staples–(e.g., Ace Clipper Model no. 702).
• Non-skid paperclips.
• Greenhouse with tables and lighting–28°C, 16 h day/25°C, 8 h night.

### Transformation Supplies

• Stirring hot plate and stir bars.
• Beakers.
• Glass flasks and sterile pouring beaker or 1 L lidded bottles for autoclaving media.
• Water bath, 55°C.
• Autoclave.
• Petri dishes–100 mm × 15 mm and 100 mm × 25 mm, sterile.
• Large, sterile Petri plate (e.g., 120 mm × 120 mm × 15 mm square plate or 150 mm × 15 mm round plate).
• 0.22 μm syringe filter.
• 0.22 μm Stericup^®^ vacuum filtration system.
• pH meter.
• Laminar flow hood.
• Sterile culture loops.
• Genesys 10S UV-VIS spectrophotometer (Thermo scientific, Waltham, MA, United States).
• Semi-micro cuvettes (Cat # 14955127, Fisher scientific, Pittsburgh, PA, United States).
• Bleach (8.25% sodium hypochlorite, e.g., Clorox).
• 70% ethanol in a spray bottle for disinfecting.
• Bead sterilizer.
• Forceps.
• Scalpel and blades.
• Handles for ears–recommend cheap, stamped sheet metal forks with all but one tine broken off.
• Embryo dissection tool (e.g., Fisher brand Handi-Hold Microspatula or Hu-Friedy plastic filling instrument #8A).
• Parafilm.
• Micropore tape.
• Plastic culture box to hold multiple plates, e.g., square plastic container with lid (31 cm × 23 cm × 10 cm, 12-5/16″ × 9-1/16″ × 4″, W × L × H, Cat # 295C), or square hinged plastic container with snap closure (33.3 cm × 9.2 cm × 5 cm, 13-1/8″ × 9-3/16″ × 2″, W × L × H, Cat # 700C) (Pioneer plastic, Dixon, KY, United States).
• 26–28°C plant tissue culture incubator (dark).
• 26–28°C plant tissue culture incubator (lighted 16 h day/8 h night, 120–150 μmol/m^2^/s).

### Molecular Analysis Supplies

• DNA extraction buffer (200 mM Tris-Cl, 250 mM NaCl, 25 mM EDTA, 0.5% sodium dodecyl sulfate).
• RNase A (DNase and protease-free).
• Chloroform.
• Isopropanol.
• 80% ethanol.
• DNase-free water.
• ExoSAP-IT™ (Applied Biosystems, Waltham, MA, United States).
• 1.5 mL centrifuge tubes.
• Scalpel and blades.
• Forceps.

### Stock Solutions

See Table 1.

**TABLE 1:** Stock solutions and preparations.*

Item	Stock	Stock solution concentration	Quantity	Dissolve in	Preparation note	Storage temp (°C)	Storage days
1	2,4-Dichlorophenoxyacetic acid (2,4-D)	0.5 mg/mL	50 mg	10 mL 1 N NaOH + 90 mL H_2_O	Dissolve 2,4-D in 10 mL 1 M NaOH, then bring the volume to 100 mL with ddH_2_O.	4	60
2	6-Benzylaminopurine (BAP)	1 mg/mL	100 mg	10 mL 1 N NaOH + 90 mL H_2_O	Dissolve BAP in 10 mL 1 M NaOH, then bring the volume to 100 mL with ddH_2_O.	4	60
3	AB buffer (20X)	20X	See Note	dH_2_O, 1 L	60 g/L K_2_HPO_4_, 20 g/L NaH_2_PO_4_	4	60
4	AB salts (20X)	20X	See Note	dH_2_O, 1 L	20 g/L NH_4_Cl, 6 g/L MgSO4⋅7H_2_O, 3 g/L KCl, 0.228 g/L CaCl_2_⋅2H_2_O	4	60
5	Abscisic acid (ABA)	0.1 mg/mL	1 mg	1 mL 1 N NaOH + 9 mL H_2_O		−20	90
6	Acetosyringone (AS)	100 mM	196.2 mg	DMSO, 10 mL	Make 10 mL stock and aliquote for single use	−20	90
7	B5H minor salts	1000X	See Note	dH_2_O, 1 L	3 g/L H_3_BO_3_, 10 g/L MnSO_4_⋅H_2_O, 0.25 g/L Na_2_MoO_4_⋅2H_2_O, 0.75 g/L KI	Room temp	60
8	Bialaphos	2 mg/mL	20 mg	dH_2_O, 10 mL		−20	60
9	Carbenicilline	100 mg/mL	1 g	dH_2_O, 10 mL		−20	90
10	Cupric sulfate	1 mg/mL	100 mg	dH_2_O, 100 mL	Make 100 mL stock	4	1000
11	Dicamba	1 mg/mL	10 mg	0.5 mL EtOH + 9.5 mL ddH_2_O	Dissolve Dicamba in 5 mL 100% EtOH, then bring the volume to 10 mL with ddH_2_O. Media containing Dicamba should store in dark.	4	120
12	Eriksson’s vitamins	1000X	See Note	dH_2_O, 100 mL	2 g/L glycine, 0.5 g/L nicotinic acid, 0.5 g/L pyridoxine⋅HCL, 0.5 g/L thiamine⋅HCL (or Phytotech E330)	4	90
13	FeSO_4_⋅7H_2_O	1.25 mg/mL	125 mg	dH_2_O, 100 mL		25	90
14	Gentamincin	50 mg/mL	5 g	dH_2_O, 100 mL		−20	90
15	Kanamycin	50 mg/mL	5 g	dH_2_O, 100 mL		−20	90
16	N6 Macronutrient stock (10X)	(for 60 mL/L)	See Note	dH_2_O, 1 L	1.66 g/L CaCl_2_⋅2H_2_O, 4.62 g/L (NH_4_)_2_SO_4_, 4 g/L KH_2_PO_4_, 1.85 g/L MgSO_4_⋅7H_2_O, 28.3 g/L KNO_3_	4	90
17	NaFe EDTA for B5H (100X)	(for 6 mL/L)	See Note	dH_2_O, 100 mL	3.7 g/L EDTA-Na_2_⋅H_2_O, 2.79 g/L FeSO_4_⋅7H_2_O	4	60
18	Nicotinic acid	1 mg/mL	100 mg	dH_2_O, 100 mL		4	120
19	Pyridoxine⋅HCl	1 mg/mL	100 mg	dH_2_O, 100 mL		4	120
20	Schenk and Hildebrandt Vitamin	100X	See Note	dH_2_O, 100 mL	100 g/L myo-inositol, 0.5 g/L nicotinic acid, 0.05 g/L pyridoxine⋅HCl, 0.5 g/L thiamine⋅HCl (or Phytotech, S826)	4	90
21	Silver Nitrate	2 mg/mL	200 mg	100 mL	Store in dark. Media containing silver nitrate should be stored in dark.	4	90
22	Spectinomycin	50 mg/mL	5 g	dH_2_O, 100 mL	If the stock is crystalized, rethaw and redissolve at 37°C before use.	−20	90
23	Thiamine⋅HCl	1 mg/mL	100 mg	100 mL	Cover with aluminum foil and keep in dark.	4	60
24	Thidiazuron (TDZ)	0.1 mg/mL	1 mg	1 mL 1 N NaOH + 9 mL H_2_O		4	90
25	Thymidine	25 mg/mL	250 mg	dH_2_O, 10 mL	If the stock is crystalized, rethaw and redisolve at 37°C before use.	4	90
26	Zeatin, trans	0.5 mg/mL	50 mg	5 mL 1 N NaOH + 95 ml ddH_2_O	Dissolve *trans-*zeatin in 5 mL 1 N NaOH, then bring volume to 100 mL with ddH2O.	4	60

*All stock solutions should be filter sterilized using 0.22 μM syringer filter or Stericup filtration system, except for item 6. Items 3 and 4 can also be sterilzed by autoclave.

### Media

See Table 2.

**TABLE 2:** Media for B104 transformation (modified from Hoerster et al., 2020).

Name	Chemical	Final conc.	Vendor/Cat info
Mother plate	glucose	5 g/L	Fisher scientific, D16
AB buffer	Bacto agar	15 g/L	BD Biosciences, 214030
	*Autoclave, cool to 55°C, then add*		
	AB buffer (20X)*	1X	
	AB salts (20X)*	1X	
	FeSO_4_⋅7H_2_O (1.25 mg/mL)*	2.5 mg/L	Fisher scientific, I146
	thymidine (25 mg/mL)*	50 mg/L	Millipore Sigma, T1895
	gentamicin (50 mg/mL)*	50 mg/L	Phytotech labs, G570
and/or	kanamycin (50 mg/mL)*	50 mg/L	Millipore Sigma, K1377
and/or	spectinomycin (100 mg/mL)*	100 mg/L	Millipore Sigma, S4014

Working plate	sodium chloride	5 g/L	Fisher scientific, S271
YEP base	yeast extract	5 g/L	Fisher scientific, BP14222
pH 6.8	peptone	10 g/L	BD Biosciences, 211677
	*Adjust pH to 6.8 with 1 M NaOH, then add*		
	Bacto agar	15 g/L	BD Biosciences, 214030
	*Autoclave, cool to 55°C, then add*		
	thymidine (25 mg/mL)*	50 mg/L	Millipore Sigma, T1895
	gentamicin (50 mg/mL)*	50 mg/L	Phytotech labs, G810
and/or	kanamycin (50 mg/mL)*	50 mg/L	Millipore Sigma, K1377
and/or	spectinomycin (100 mg/mL)*	100 mg/L	Millipore Sigma, S4014

Infection	MS basal salt mixture	4.33 g/L	MilliporeSigma, M5524
700A	myo-inositol	0.1 g/L	MilliporeSigma, I3011
pH 5.2	nicotinic acid (1 mg/mL)*	0.5 mg/L	MilliporeSigma, N0761
	pyridoxine⋅HCl (1 mg/mL)*	0.5 mg/L	MilliporeSigma, P8666
	thiamine (1 mg/mL)*	10 mg/L	MP biomedicals, 194749
	casamino acids	1 g/L	Fisher scientific, BP1424
	sucrose	68.5 g/L	Fisher scientific, BP220
	glucose	36 g/L	Fisher scientific, D16
	2,4-Dichlorophenoxyacetic acid (2,4-D) (0.5 mg/mL)	1.5 mg/L	MilliporeSigma, D7299
	*Adjust pH to 5.6 with 1 M NaOH*		
	*Filter sterilize (0.22 μM)*		
	Add thymidine (25 mg/mL)* freshly before use	50 mg/L	Millipore Sigma, T1895
	Add acetosyringone (AS) (100 mM)* freshly before use	100 μM	Millipore Sigma, D134406

Co-cultivation	MS basal salt mixture	4.33 g/L	MilliporeSigma, M5524
710I	myo-inositol	0.1 g/L	MilliporeSigma, I3011
pH 5.6	nicotinic acid (1 mg/mL)*	0.5 mg/L	MilliporeSigma, N0761
	pyridoxine⋅HCl (1 mg/mL)*	0.5 mg/L	MilliporeSigma, P8666
	thiamine (1 mg/mL)*	10 mg/L	MP biomedicals, 194749
	proline	0.7 g/L	Alfa Aesar, A10199
	sucrose	20 g/L	Fisher scientific, BP220
	glucose	10 g/L	Fisher scientific, D16
	2-(*N*-morpholino)ethanesulfonic acid (MES)	0.5 g/L	Fisher scientific, BP300
	2,4-D (0.5 mg/mL)*	2 mg/L	MilliporeSigma, D7299
	*Adjust pH to 5.6 with 1 M NaOH, then add*		
	agar	8 g/L	MilliporeSigma, A7921
	*Autoclave, cool to 55°C, then add*		
	acetosyringone (AS) (100 mM)*	100 μM	Millipore Sigma, D134406
	thymidine (25 mg/mL)*	50 mg/L	Millipore Sigma, T1895
	silver nitrate (2 mg/mL)*	1 mg/L	Fisher scientific, S181

Resting	MS basal salt mixture	4.33 g/L	Phytotech labs, M5605 (11 g/L)^#^
605G	N6 macronutrient stock (10X)*	0.6X	Phytotech labs, M5605 (11 g/L)^#^
pH 5.6	B5H Minor salts (1000X)*	0.6X	Phytotech labs, M5605 (11 g/L)^#^
	NaFe EDTA for B5H (100X)*	0.6X	Phytotech labs, M5605 (11 g/L)^#^
	Eriksson’s vitamins (1000X)*	0.4X	Phytotech labs, M5605 (11 g/L)^#^
	Schenk and Hildebrandt vitamins (100X)*	0.6X	Phytotech labs, M5605 (11 g/L)^#^
	potassium nitrate	1.68 g/L	Phytotech labs, M5605 (11 g/L)^#^
	thiamine HCl (1 mg/mL)*	0.2 mg/L	Phytotech labs, M5605 (11 g/L)^#^
	proline	2 g/L	Phytotech labs, M5605 (11 g/L)^#^
	sucrose	20 g/L	Fisher scientific, BP220
	glucose	0.6 g/L	Fisher scientific, D16
	casein hydrolysate	0.3 g/L	Thermo fisher scientific, J12855-P2
	2,4-D (0.5 mg/mL)*	0.8 mg/L	MilliporeSigma, D7299
	*Adjust pH to 5.6 with 1 M NaOH, then add*		
	TC agar	6 g/L	Phytotech labs, A296
	*Autoclave, cool to 55°C, then add*		
	dicamba (1 mg/mL)*	1.2 mg/L	Phytotech labs, D159
	silver nitrate (2 mg/mL)*	3.4 mg/L	Fisher scientific, S181
	carbenicilline (100 mg/mL)*	100 mg/L	Phytotech labs, C346

Maturation	MS basal salt mixture	4.33 g/L	MilliporeSigma, M5524
13329B	cupric sulfate (1 mg/mL)*	1.25 mg/L	MilliporeSigma, C2857
pH 5.6	myo-inositol	1 g/L	MilliporeSigma, I3011
	proline	0.7 g/L	Alfa Aesar, A10199
	sucrose	60 g/L	Fisher scientific, BP220
	zeatin, *trans* (0.5 mg/mL)*	0.5 mg/L	Phytotech labs, Z125
	*Adjust pH to 5.6 with 1 M NaOH, then add*		
	agar	7 g/L	MilliporeSigma, A7921
	*Autoclave, cool to 55°C, then add*		
	abscisic acid (ABA) (0.1 mg/mL)*	0.1 mg/L	MilliporeSigma, 862169
	6-Benzylaminopurine (BAP) (1 mg/mL)*	1 mg/L	MilliporeSigma, B3408
	thidiazuron (TDZ) (0.1 mg/mL)*	0.1 mg/L	Phytotech labs, T888
	carbenicilline (100 mg/mL)*	100 mg/L	Phytotech labs, C346
11329B3	bialaphos (2 mg/mL)*	3 mg/L	Gold Biotechnology, B0178
11329B6	bialaphos (2 mg/mL)*	6 mg/L	Gold Biotechnology, B0178

Rooting	MS basal salt mixture	4.33 g/L	MilliporeSigma, M5524
13158B2	myo-inositol	0.1 g/L	MilliporeSigma, I3011
pH 5.6	sucrose	40 g/L	Fisher scientific, BP220
	*Adjust pH to 5.6 with 1 M NaOH, then add*		
	Bacto agar	7 g/L	BD Biosciences, 214030
	*Autoclave, cool to 55°C, then add*		
	bialaphos (2 mg/mL)*	2 mg/L	Gold Biotechnology, B0178

*See Table 1 for stock solution preparation.

*^#^Pre-made 605 medium salts is available at Phytotech labs (M5605) or add the ingredients described.*

## Methods

### Growing B104 Plants to Produce Immature Embryos for Transformation

**FIGURE 2:**
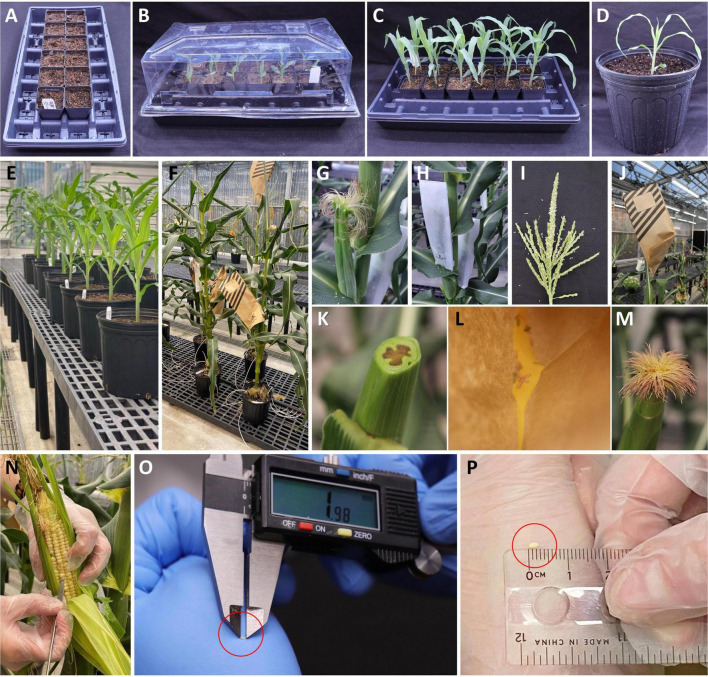
Growing maize donor plants for immature embryo production. **(A)** Standard flat with small pots filled with potting mix for planting maize seed. **(B)** Six-day-old germinated seedlings under a humi-dome. **(C)** Two-week-old seedlings ready to be transplanted into large pots. **(D)** A seedling transplanted to a large pot. **(E)** Plants on a 1 m tall bench. **(F)** Pollinated mature B104 plants on the floor. **(G)** An un-pollinated B104 ear (female flower) with emerged silks. **(H)** A shoot bag covering an un-pollinated B104 ear. **(I)** A mature tassel (male flower) ready to be used for pollination. **(J)** A pollination bag covering a mature tassel for the purpose of collecting fresh pollen. **(K)** An un-pollinated B104 ear with cut silks. **(L)** Freshly collected pollen in a tassel bag. **(M)** Freshly pollinated silks, 1 day after silks were cut. **(N)** Extracting an immature embryo for measurement while the ear is still growing on the plant. **(O)** Measurement of an embryo size with a caliper. **(P)** Estimate of an embryo size with a ruler.

1. Maize B104 plants are grown in a greenhouse equipped with artificial lighting and reverse osmosis (RO) water systems in the Donald Danforth Plant Science Center (St. Louis, MO, United States). The greenhouse conditions are as follows: photoperiod, 14/10 h (day/night); temperatures, 28/22°C (day/night); relative humidity, 40/50% (day/night); Supplemental light intensity, 150 μmol/m^2^/s at 1.5 m (5 ft) above ground. Lighting is a mixture of 50% of 1000 W Metal Halide and 50% of 1000 W High-Pressure Sodium Fixtures mounted 4 m (13 ft) above ground.
2. Maize seeds are usually sown in small containers filled with commercial potting mix. Plastic inserts (1801 deep inserts) are filled with Metromix 360 potting mix and placed onto STF-1020-OPEN flat with drain holes (Figure 2A). Before sowing, the potting mix must be wetted thoroughly but not excessively.
3. B104 seeds (one per cell) are placed in the potting mix approximately 2.5 cm (1 inch) deep from the surface. The tray is covered with a plastic humi-dome (Figure 2B) and placed on a bench in the greenhouse with the conditions described above. In the case of needing year-long donor plant production, seeds can be sown every 3–4 days, depending on growth space availability.
4. Soil moisture is monitored on daily basis. Sprouting seeds can be expected about 4 days after the sowing. Keep the humi-dome on the flat until the sixth day after the planting. Seedlings are watered only if the potting mix appears to be dry.
5. Seedlings with ∼4–5 leaves can be transplanted into large pots approximately 2 weeks after the sowing (Figure 2C).
6. Potting mix Pro-Mix BRK20 is used to fill 2.5 gallons (9.4 L) nursery pots. Each pot is top-dressed with 2/3 tsp tomato maker, 2/3 tsp Osmocote, and 1/2 tsp Sprint Iron chelate. Then RO water is used to water the pot until the potting mix becomes saturated.
7. Seedling, with soil adhering to the roots, is carefully transplanted from a small plastic insert into the large pot with properly prepared and wetted potting mix (Figure 2D) and placed on a 1 m tall bench (about 2.7 m or 9 ft below the lights) for about 2 weeks (Figure 2E). Plants can be moved to the floor 2–3 weeks after the transplanting (Figure 2F) and spaced at least 12.5–15 cm (5–6 inches) apart between pots.
8. Plants are watered as needed. Overwatering of maize plants will result in poor development of the root system. Maize plants grow differently under different growth conditions. In the greenhouse described in this work, the B104 plants are watered daily with fertilizer solution (Jacks 15-5-15 at 400 ppm, pH 6.0). Every third watering is with RO water. Watering frequency is 1–2 times daily as needed.
9. Female flower (silk) emergence (Figure 2G) occurs approximately 60 days after the sowing. To ensure controlled pollination, emerging ears are covered with a wax-treated paper shoot bag prior to silk emergence (Figure 2H). To avoid pollen cross-contamination, plants and shoot bags should be closely monitored. Any uncovered or improperly covered female flowers should be discarded.
10. Male flower (tassel) emergence (Figure 2I) should occur around the same period, although not always synchronized with the female flowers. The tassel should be covered by a brown pollination bag (Figure 2J) once it starts to produce pollen.
11. To produce immature embryos, silks are pollinated using pollen from the same plant or a sibling B104 plant. To encourage silks growth, the ear shoot tip can be cut (Figure 2K) one day prior to the pollination. Using a pair of clean scissors, cut the female flower ∼2.5 cm (1 inch) from the top of the shoot. The cut ear is immediately covered by a shoot bag and allowed to grow for another day to ensure freshly grown, evenly distributed silks are used for pollinations. Before being used to cut the next ear shoot, scissors should be sanitized with 70% ethanol to prevent fungal or bacteria contamination carryover between ears.
12. Pollination should take place in the mid-morning the next day. Pollen is collected from a B104 plant with a bagged tassel. To release pollen from anthers, the tassel bag, and the tassel should be shaken by hand gently, the bag with the pollen is then removed from the tassel for pollination (Figure 2L). The tassel is immediately covered by another new tassel bag if the tassel is to be reused in next day. Otherwise, the tassel should be removed from the plant (detassel) to avoid any unwanted pollen shedding and cross-contamination.
13. Identify a bagged ear shoot that was cut the previous morning. Remove the shoot bag, quickly but carefully sprinkle the pollen evenly onto the freshly grown silks (Figure 2M). The pollinated ear is immediately covered with a brown pollination bag that is labeled with the cross information such as plant ID, date, and nature of the cross (Figure 2F). Pollen collected from one tassel with 50–70% opened anthers can be used to pollinate multiple ear shoots. Pollen from freshly opened anthers is most desirable, although B104 pollen of up to 4 days post-anther emergence can still be used.
14. Immature embryos of 1.8–2.0 mm (up to 2.5 mm) in size are ideal for maize transformation. To obtain the embryos of the appropriate sizes, ears should be checked for embryo development while they are still on the plant (Figure 2N) around 10 days after the pollination. Embryo sizes can be measured by a caliper (Figure 2O) or estimated by a small ruler (Figure 2P). Typically, ears can be harvested between 10 and 14 days depending on the temperature of the greenhouse, which may be impacted by exterior weather conditions. In general, embryos reach desired sizes faster in the summer season than in the wintertime.

*Important: Plants should be monitored routinely throughout their life for insect and fungal pathogens and should be appropriately treated when pathogens are present. Plants that have been kept free of pathogens and received the minimum possible pesticide applications to produce the healthiest embryos for transformation.*

### Preparation of *Agrobacterium* Plate Cultures

**FIGURE 3:**
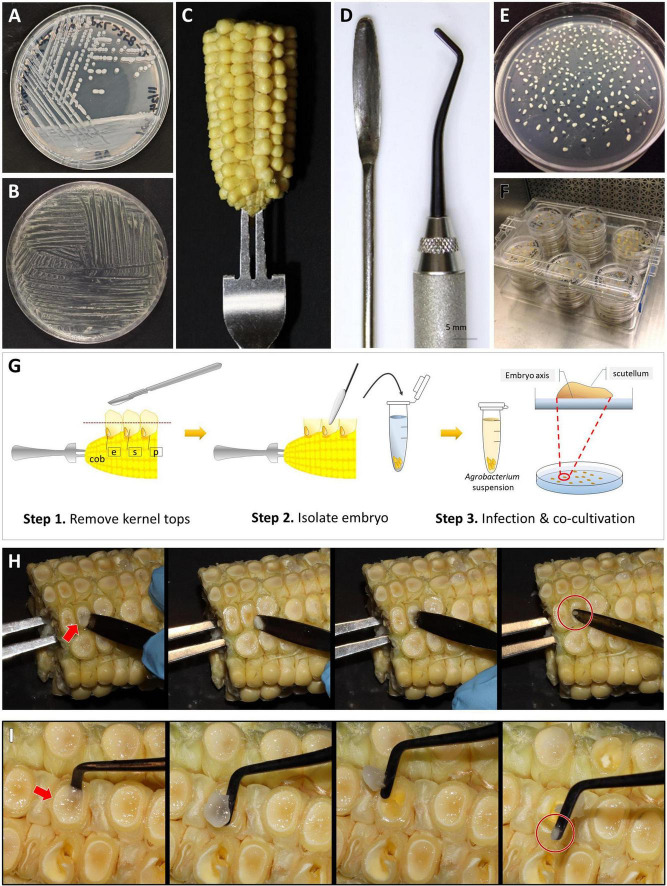
Embryo dissection, infection, and co-cultivation. An *Agrobacterium* “mother” plate **(A)** and “working” plate after 18 h incubation, streaked by a disposable loop **(B)**. **(C)** A B104 ear halves on a handle made of a fork. **(D)** Tools for embryo dissection; a micro spatula (left) and a dental filling instrument (right). **(E)** A co-cultivation plate with dissected immature embryos. All embryos were re-oriented scutellum side up. **(F)** A plastic culture box filled with plates. **(G)** Cartoon illustration of the embryo dissection and infection process. Step 1, remove kernel tops using a sterile scalpel; Step 2, isolate embryo from the kernel and transfer it to a tube filled with liquid 700A infection medium; Step 3, after infection, place embryo onto co-cultivation medium and orient the embryos by placing them scutellum side up. e: embryo, s: endosperm, and p: pericarp. Demonstration of embryo dissection using a micro spatula **(H)** or a dental filler **(I)**. The arrows indicate the embryo side of the kernel. Circles highlight isolated embryos.

1. At least three days before the infection or earlier, prepare a “mother” plate (Table 2) of *Agrobacterium* strain KL2013vir/thy from a glycerol stock that was originally prepared from a single colony. Streak *Agrobacterium* on the “mother” plate using a sterile loop.
2. Incubate the mother plate at 28°C in the dark for 36∼48 h. The “mother” plate with fully grown *Agrobacterium* colonies (Figure 3A) can be stored at 4°C in the dark for up to 7 days.
3. Day 0: One day before the embryo infection experiment, prepare a “working” plate (Table 2) of KL2013vir/thy. Collect 7–8 colonies (2 mm diameter) from the “mother” plate (Figure 3A) and streak them evenly on the “working” plate surface. The purpose of collecting at least 7–8 colonies from the mother plate is to ensure that sufficient *Agrobacterium* cells are inoculated on the “working” plate. An L-shaped spreader can be used to evenly spread the cells on the plate. To ensure enough *Agrobacterium* culture for infection, prepare one or two additional “working” plates as back-ups.
4. Incubate the “working” plates at 28°C in the dark for 16–20 h (Figure 3B). Avoid using “working” plates that are older than 24 h after the streaking.

### Ear Sterilization and Embryo Dissection (Day 1, Stage 1)

1. Remove the husk leaves and silks from the ears harvested from the greenhouse. Insert an ethanol-sterilized handle into either the top or the base of the ear (Figure 3C). The ear can be cut into two halves horizontally to fit into a large container for sterilization.
2. Place the ear into a large, sterile beaker on a laminar flow bench. Pour sterilization solution [2 L of 20% commercial bleach (8.25% sodium hypochlorite) plus two drops of Tween 20] into the beaker so that the solution will cover the ear completely. Gently swirl the ears and tap them to the beaker bottom 10–20 times to remove bubbles trapped on the surface of the ears. Keep the ear in the sterilization solution for 30 min, stirring lightly every 10 min to ensure all ear surfaces are in contact with the solution.
3. Discard the sterilization solution into a large waste container. Add a large volume of autoclaved Millipore water into the beaker to wash the ears. Stir lightly 2–3 times and leave the ears in the water for 5 min. Repeat the wash step two more times, for 2 min each to complete the sterilization step.
4. Place the sterilized ear on a large, sterile Petri plate on the laminar flow bench. Using a sharp, clean scalpel, carefully remove the opaque kernel top, 2∼3 mm thickness at once until the clear endosperm is exposed (Figure 3G). Cutting too deep into the kernel may accidentally damage the embryos, especially when they are larger than 2 mm.
5. Insert a sterile micro-spatula (Figure 3D) perpendicular to the ear axis at the bottom of the kernel then squeeze gently toward the ear-tip side (Figures 3G,H). The embryo is located at the bottom, ear-tip side of the kernel. The embryo will emerge between the pericarp and endosperm. If the embryo does not pop up from the kernel, gently scoop out the endosperm with the spatula to isolate the embryo from the adhering tissue. Figure 3I demonstrates the use of a dental filling instrument for embryos isolation. The micro-spatula can be used for squeezing or scooping, while the dental filling instrument is more efficient in scooping the immature endosperm.
6. Put the embryo into a 2 mL Eppendorf tube containing 1.6 mL of liquid 700A infection medium without thymidine and acetosyringone (AS, Table 2). Collect up to 100 embryos in one tube (Figure 3G, Step 2). The embryos can be stored in the tube on the bench for 2–3 h.

### *Agrobacterium* Infection and Co-cultivation (Day 1, Stage 2)

1. In a 50 mL conical tube containing 10 mL of 700A infection medium (Table 2), add 20 μL of 25 mg/mL thymidine (final concentration, 50 mg/L) and 10 μL of 100 mM AS (final concentration, 100 μM). Thymidine and AS should be added freshly before each experiment. Divide 700A infection medium into two 50 mL tubes (5 mL each).
2. Harvest *Agrobacterium* cells from the working plate (Figure 3B) using a sterile loop. Inoculate the bacteria in the 5 mL 700A infection medium. Vortex the tube for 4–5 s to fully resuspend the bacteria.
3. Measure optical density (OD) of *Agrobacterium* suspension at 550 nm on a spectrophotometer and adjust the OD_550_ of the suspension to 0.50 ± 0.05. This *Agrobacterium* suspension should be used immediately as it can readily form aggregates within 30 min after inoculation.
4. Before infection, wash the isolated embryos once with a freshly prepared 700A infection medium. Carefully remove the 700A infection medium using a pipet, then add 1 mL of freshly made *Agrobacterium* suspension to the embryos in the tube. Cap the tube and gently invert it 3–4 times. Incubate the tube at room temperature (22–24°C) for 5 min, by placing it sideways on the bench.
5. Tap the tube 4–5 times to dislodge the embryos from the sidewalls of the Eppendorf tube. Then pour the bacteria suspension containing the embryos onto a co-cultivation plate (710I, Table 2). Tilt the plate to collect and remove as much bacterial suspension as possible with a pipette.
6. Under a dissecting microscope in the laminar flow bench, carefully orient the embryos by placing them scutellum side (smooth and round side) up (Figure 3G, Step 3). Use the micro spatula or the dental filling instrument scaler (Figure 3D) to gently flip the embryos; avoid damaging the embryos (Figure 3E).
7. Wrap the plates with a micropore tape or place the unwrapped plates in a clean plastic culture box (Figure 3F). Incubate the plates at 20°C in the dark for 16–20 h.

### Resting (Day 2), Maturation and Selection (Day 9)

1. Transfer and place the embryos, scutellum side up, to a resting medium (605G, Table 2). Place no more than 25–30 embryos per plate. Incubate the plates at 28°C in the dark for 7 days. Transient RFP expression is visible 3 days post-infection.
2. Eight days after infection, transfer the embryos to a maturation medium supplemented with 3 mg/L bialaphos (13329B3, Table 2). Carefully remove growing hypocotyls with forceps and scalpel. This is to prevent the hypocotyl from lifting or re-orienting the embryo away from the medium. Place no more than 8–10 embryos per plate. Incubate the plates at 28°C in the dark for 10 days. Growing callus tissue emerges in this stage.
3. Subculture the callus pieces to a new maturation medium containing 6 mg/L bialaphos (13329B6, Table 2). If the calli grow vigorously (over 2 cm^2^), place 4–5 calli per plate. The base of calli with growing shoots may break naturally. If it breaks apart, spread the growing shoots onto the medium. Incubate the plates at 28°C in the dark for 14 days.
4. The RFP gene mCherry expression in the callus or developing shoots can be examined by using either a fluorescent microscope (Olympus SZH10 stereo microscope with Texas red filter, excitation wavelength 535–585 nm, emission wavelength 605–690 nm) or using a NIGHTSEA dual fluorescent protein flashlight and filter glasses (NIGHTSEA LLC, Lexington, MA, United States). Tiffen 29 dark red filter can be used for DSLR camera for imaging.

### Rooting (Day 33)

**FIGURE 4:**
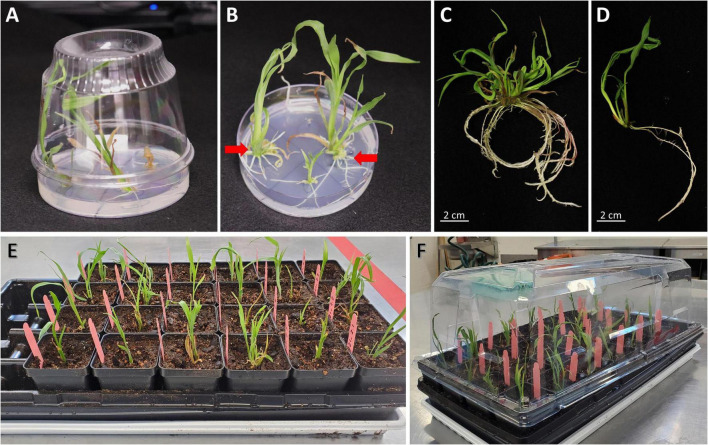
Regenerated T0 plantlets from rooting stage to acclimatization in soil. **(A)** Plantlets growing on the rooting medium. Sundae cup is used as a cover to give vertical space to grow. **(B)** Regenerated plantlets ready to be moved to potting mix. Red arrows indicate plantlets with healthy roots. **(C)** Plantlet representing bushy phenotype or multiple shoot cluster. **(D)** Single-shoot plantlet ready to be transplanted to potting mix. **(E)** T0 regenerated plantlets transplanted to potting mix. **(F)** A plant propagation tray covered with a humi-dome to aid in acclimatization.

1. Transfer callus pieces with fully developed shoots onto a rooting medium containing 2 mg/L bialaphos (13158B2, Table 2). Place one or two callus pieces in one plate to ensure enough space for shoot growth. Remove excessive callus materials around the shoots. Incubate the plates at 26°C in a light chamber, with a photoperiod of 16 h light and 8 h dark (80 μmol/m^2^/s) for 14 days.
2. Plantlet (>4 cm) with developed roots (>2–3 cm) (Figures 4A,B) can be transplanted to soil and moved to the growth chamber for acclimatization. Green mature shoots without roots may be subcultured for an additional 14 days under the same condition. The excessive callus materials associated with the shoots should be removed during the subculture.
3. If the T0 plantlets are to be brought to maturity for seeds, pollen donor plants will need to be planted at this time or 60 days prior to the projected date of pollination. This step needs to be taken to ensure access to high-quality pollen. Because female and male flowers of *in vitro* regenerated maize plants are often not synchronized, wild-type B104 plants can be used as pollen donors to pollinate transgenic T0 female flowers. When the root of the T0 plant is visible in the Petri dish, germinate B104 seeds every 5–7 days until all transgenic plantlets are transplanted from Petri dishes to the soil.

### Transplantation and Acclimatization (∼Day 50)

1. When T0 plantlets have established in tissue culture rooting media, they are ready to be transplanted to soil. Transplant plantlets with healthy roots (longer than 2–3 cm, as shown by red arrows in Figure 4B). Smaller plants can be subcultured to fresh medium for continued growth.
2. Fill 1801 deep inserts (2/3 of the height) with autoclaved potting mix such as Metromix 360 and place them into STF-1020-OPEN flat with drain holes (Figure 2A). Make a hole for the plant in the middle of each cell using a spoon. Autoclaved potting mix is recommended to prevent fungal contamination. The autoclaved moisten potting mix (60∼70% saturated) can be stored in a cool place for 2 weeks.
3. Using a pair of sterile forceps, first break up the media around the roots on the plate, then hold the base of the plantlet, gently pull it from the gel media without damaging the roots.
4. Hold the plantlet with a gloved hand and gently swirl it in sterile water 3–4 times to wash off the plant medium. It is important to remove excess medium associated with the roots as the presence of gel may bring fungal growth, which can kill the young plant. However, it is also important to avoid any physical damage to the plants during the process. If the roots are coated with plant medium, do not scrape it off, but rather gently rinse to remove the remaining media clumps.
5. Carefully remove any callus and shoots without roots using a pair of sterile forceps. Non-regenerated tissue will naturally come off from the regenerated plantlets. If the tissue is tightly bound with the plantlet, do not use a scalpel to dislodge them from the plantlet. For bushy plants (Figure 4C) or multiple shoot clusters, carefully separate individual plants from the cluster as much as possible but avoid tearing or damaging roots or shoots in the process. The cleaned individual plantlet (Figure 4D) can be examined for the red fluorescent protein expression on the root at this stage.
6. Place one plantlet per cell. Bury the roots and base of stem 3–5 cm deep in the potting mix (Figure 4E). Cover the flat with a plastic humi-dome (Figure 4F). Keep the plantlet upright to avoid touching the side of the humi-dome. Place the tray in a growth facility with 14/10 h (day/night) photoperiod, and 28/22°C (day/night) temperature. Plantlets transferred from the tissue culture media to potting mix tend to desiccate quickly due to low humidity. Therefore, the use of a humi-dome is critical to maintaining the moderate humidity level for the optimal acclimation to greenhouse growth conditions.
7. Check the plantlets and soil moisture daily. Water only when the soil becomes dry.
8. Four to six days after transplanting, remove the plastic humi-dome and continue watering as needed.
9. Phenotyping and genotyping of the T0 plantlets can be performed approximately 10–12 days after the transplantation (see below sub-section).
10. After approximately 2 weeks, healthy and robust-looking plantlets can be transplanted into a large pot as described in steps 6–8 in the above section of “Growing donor plants for the production of the immature embryo for transformation.” Both 1 gallon (3.8 L) and 2.5 gallon (9.4 L) pots can be used for growing maize plants to full maturity. It is critical not to overwater the plants, especially when they are young.

### T0 Seed Production

1. When ears start to emerge (50–100 days post-transplant), T0 plants are pollinated to produce seed for subsequent generation testing and analysis. Male and female flower development can occur at separate times on an individual T0 plant, which will necessitate crossing B104 wild-type or other transgenic B104 plants to generate seeds.
2. To avoid pollen cross-contamination, transgenic plants should not be grown with any non-transgenic plants in the same growth room. All emerging ears are covered with a wax-treated paper shoot bag to prevent pollen contamination of the silks before pollination.
3. Cover the tassel with a bag. The bag should be folded to secure it around the stem with a paper clip to prevent pollen from escaping (Figure 2J).
3. If the greenhouse space is crowded, detasseling the T0 plants will be necessary to prevent transgenic pollen cross-contamination. When the tassel is extended from the whorl, pull it out gently for detasseling. The detasseling may stimulate shoot growth.
4. Follow steps 9–13 described in the section “Growing donor plants for the production of the immature embryo for transformation” for T0 plant pollination.
5. Immediately cover the pollinated ear with the pollination bag. Record the ear donor, pollen donor, silking date, and pollination date on the tassel bag. Staple the back of the tassel bag.
6. Ten days post-pollination, remove the pollination bag and slide it into the node between the ear and the plant’s stalk. Peel back the husk to observe the seed set and allow fresh air to the ear to prevent fungal contamination.
7. Thirty days post-pollination, stop watering to allow dry down and senescence of plants with mature ears. Keep the tassel bag on the stalk to keep the record.
8. Forty days post-pollination, harvest mature dried T0 ear and place in pollination bag, record the harvest date on the bag.
9. Dry the ears at room temperature inside the pollination bag on a benchtop for 2–3 weeks.
10. Remove kernels from ear and place in a seed packet, label the packet with appropriate experimental information, harvest date, and seed weight.
11. Seeds can be stored in a cool and dry place (15°C, Relative Humidity < 30%). For long-term storage, seeds can be kept at −20°C with an additional desiccant such as silica gel.

### Evaluating of T0 Plants for *glossy2* Phenotype

1. Knockout mutants of maize *glossy2* (*Gl2*) gene (Bianchi et al., 1975) can be evaluated on T0 plantlets. Although the gene product of *Gl2* is still unknown, it is responsible for the formation of a hydrophobic waxy cuticle layer in juvenile leaf tissues. The loss-of-function mutants can be readily identified by misting water on the young leaf surface (Bianchi et al., 1975). Water will roll off from wild-type leaf, whereas *gl2* null mutations affect the deposition of extracellular cuticular lipids allowing water droplets to adhere to the leaf surface. This is most obvious when working with homozygous or biallelic mutant plants.

*Note that misting on T0 plants can only be used as a preliminary evaluation. It should not be used to make conclusion on whether the plant is a loss-of-function mutant event. Sequencing analysis is necessary to verify and confirm the phenotypes observed on the T0 plants.*

2. Plants that have stayed out of the humi-dome at least for 4–6 days (typically 10 days post-acclimatization) can be evaluated for *gl2* phenotype. Use a spray bottle to mist the leaf surface with tap water.
3. The *gl2* loss-of-function mutants would show water droplet adherence on both sides of the leaf blades while the surface of wild-type leaf blades would repel water droplets due to the hydrophobic cuticle layer.

### Molecular Analysis of T0 Plants

1. Total genomic DNA can be isolated from the transgenic maize plants using a modified version of the protocol described by Edwards et al. (1991). Sample 3∼4 cm^2^ of fresh leaf tissue in a 1.5 mL tube. Add 500 μL DNA extraction buffer containing 100 μg/mL RNase A into each tube and grind the leaf tissue using a polypropylene homogenizing pestle attached to an electric drill. Incubate the tubes for 5–10 min at room temperature. If the leaf tissue is smaller than 2 cm^2^, reduce the DNA extraction buffer to 250 μL.
2. Add 500 μL (equal volume to the DNA extraction buffer used in step 1) of chloroform and mix gently by inverting the tubes for 3 min, and centrifuge for 5 min at a maximum speed using a benchtop centrifuge (21,130 × *g*).
3. Carefully transfer about 300 μL of the top aqueous phase to a new tube and add 240 μL (80% of aqueous phase, v/v) of isopropanol. If 250 μL of extraction buffer was used, then transfer 150 μL of the aqueous phase into a new tube and add 120 μL of isopropanol. Be careful not to take any interphase or organic phase into the new tube. Mix well by inverting 4–5 times and centrifuge for 5 min at a maximum speed (21,130 × *g*).
4. Remove the supernatant using a 1 mL pipette and rinse the genomic DNA pellet once with 500 μL of ice-cold 80% ethanol.
5. Centrifuge for 2 min at a maximum speed on a benchtop centrifuge and carefully remove the ethanol using a 1 mL pipette. Briefly spin down the tubes to collect the remaining ethanol on the tubes and remove it using a 200 μL pipette. Air-dry the genomic DNA pellets for 10 min on a flow bench.
6. To redissolve the genomic DNA pellet, add 50 μL of nuclease-free water and finger tap the tubes 4–5 times. Measure the genomic DNA concentration using a Nanodrop spectrophotometer. Typically, 20–200 ng/μL of genomic DNA is obtained using this method (1–10 μg of total genomic DNA), and 1 μL of genomic DNA is used for a 20 μL PCR reaction.
7. Perform PCR screening to identify transgenic plants and to genotype the target gene, *Gl2*. PCR amplifies ∼1 kb fragments from the *gl2* locus and the *Cas9* gene using the primer sets and PCR conditions previously reported (Lee et al., 2019).
8. Run 1% agarose gel electrophoresis to resolve the PCR products and identify *Cas9*-positive T0 plants.
9. For the *Cas9*-positive plants, treat the PCR products from the *gl2* locus with the ExoSAP-IT™ reagent for Sanger sequencing analysis. Mix 5 μL of *gl2* PCR product with 2 μL ExoSAP-IT™ reagent and incubate 30 min at 37°C followed by incubation for 15 min at 80°C for enzyme inactivation.
10. Perform Sanger sequencing using a preferred service provider.
11. Analyze the sequence trace files from the transgenic plants and wild-type B104 using publicly available web-based CRISPR editing analysis software; Tracking of Indels by Decomposition (TIDE; Brinkman et al., 2014), Inference of CRISPR Edits (ICE; Hsiau et al., 2019), and Degenerate Sequence Decode (DSDecodeM, Xie et al., 2017). Short indel mutations can be readily identified by these programs and it is recommended to use at least two software to cross-check the outcomes.

*Note: Direct Sanger sequencing of the PCR products and trace file analyses using online decode tools demonstrated here are recommended for rapid screening of mutant events. For precise/final genotyping analysis, cloning of the PCR products into a plasmid vector and then Sanger sequencing of multiple clones are recommended.*

## Results

### Transient and Stable Transformation

**FIGURE 5:**
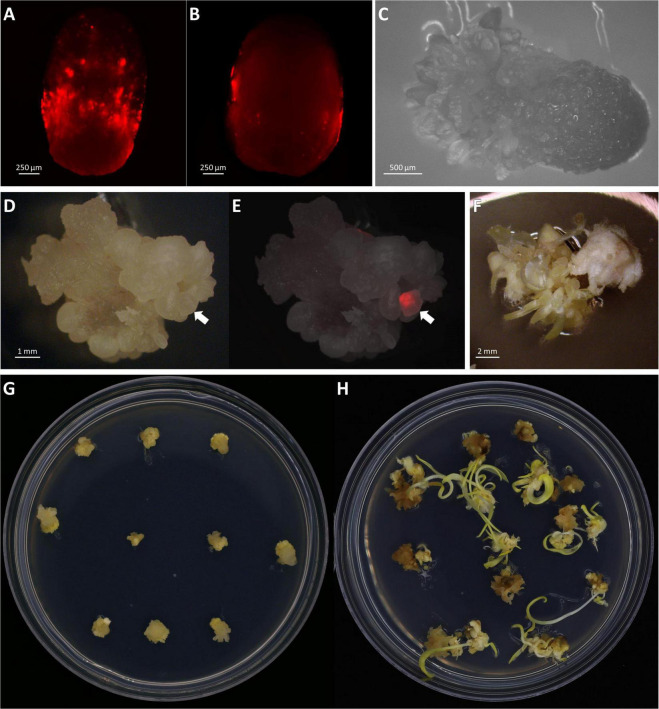
Transient and stable transformation. Observation of transient RFP expression 3 days post-infection on the middle of the scutellum **(A)** or the side of the embryos **(B)**. **(C)** Somatic embryogenesis on embryo scutellum side, 8 days post-infection in bright field. Observation of stable transformation and callus formation of maize embryo under bright field **(D)** and RFP field overlayed **(E)**. The arrows indicate callus with the RFP expression. **(F)** Tentacle-like structure on a callus during the maturation stage, 21 days post-infection. Maize callus on the maturation I medium, 18 days post-infection **(G)** and the maturation II medium, 31 days post-infection **(H)**.

In this work, three independent transformation experiments were conducted and a total of 363 embryos collected from 11 ears were infected (Table 3). The binary vector construct pKL2013 (Figure 1A) used in this work carries a red fluorescent protein (RFP) mCherry gene, which is under a constitutive promoter P35S. This visual marker gene was used to monitor the T-DNA delivery and transient gene expression after infection and co-cultivation. Nearly all infected embryos showed transient RFP expression 3 days post-infection (Figures 5A,B). RFP expression patterns can be various; they can be found on the middle of the scutellum (Figure 5A) or the side of the embryos (Figure 5B).

**TABLE 3:** Summary of transformation frequency (TF) of three experiments.

EXP	Nr. of ears	Nr. of infected Embryos	Nr. of regenerants	% regenerants	Nr. of T0 event	% TF
ALT1-CAS	3	110	38	34.5%	10	9.1%
ALT2-CAS	5	111	11	9.9%	2	1.8%
ALT3-CAS	3	142	23	16.2%	12	8.5%
**Total**	**11**	**363**	**72**	**19.8%**	**24**	**6.6%**
**Average**	**3.7**	**121**	**24**	**20.2%**	**8**	**6.4%**

A total of 72 shoots were regenerated from infected embryos that were grown on the bialaphos-containing selection media. The regeneration rates ranged from 9.9 to 34.5% (Table 3) with an average of 20.2%. PCR analysis was performed on all 72 plants and 24 of them (33.3%) were transgenic carrying the *Cas9* gene. The transformation frequency (TF, number of T0 plants per 100 infected embryos) of the three infection experiments using this protocol ranged from 1.8 to 9.1% with an average of 6.4% (Table 3), which was comparable to the 4% TF reported previously (Raji et al., 2018).

### Plant Regeneration

A major improvement in this protocol is the significant reduction in the time it takes to produce transgenic plants. In this work, rooted transgenic T0 plantlets were obtained in as little as 50 days after infection (Table 4). Compared to the conventional B104 protocol (Raji et al., 2018), this method reduces callus selection and the proliferation timeline from 161 to 42 days (Table 4, Steps 4–9). After resting (8 days post-infection), somatic embryos started to emerge on the scutellum of the embryos (Figure 5C). These embryos were transferred to a maturation medium containing 3 mg/L bialaphos (13329B3, Table 2). At the beginning of the maturation stage, developing tissues form enlarged somatic embryo-like structures which are similar to Type I callus described by Wan et al. (1995) (Figures 5D,E,G). After 10–14 days on the maturation medium (18–22 days post-infection), the callus formed white and opaque tentacle-like tissues (Figure 5F). The tentacle-like tissues further developed to pale-yellow shoots (Figure 5H). To suppress non-transgenic shoot growth, the developing callus tissues were moved to a maturation medium containing 6 mg/L bialaphos (13329B6, Table 2).

**TABLE 4:** Comparison of conventional and improved B104 transformation methods.

Step	Activities	Day of Action	
		Conventional^1^	Improved^2^
1	Agro strain preparation	Day 0	Day 0
2	Embryo dissection, infection, co-cultivation	Day 1	Day 1
3	Resting	N/A	Day 2
4	Shoot formation and selection I	Day 4	Day 9
5	Shoot formation and selection II	Day 18	Day 19
6	Bulking	Day 60	N/A
7	Regeneration and selection	Day 137	N/A
8	Rooting	Day 151	Day 33
9	Moving to soil	Day 165	Day 51

*^1^Based on Raji et al. (2018).*

*^2^This work.*

**FIGURE 6:**
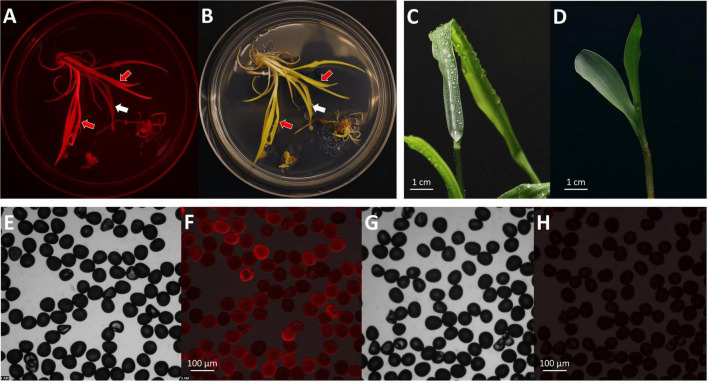
Phenotypes of T0 transgenic plants. Observation of mature shoots with roots on the maturation II medium under an RFP channel **(A)** and a bright field **(B)**. Black arrow indicates a shoot with RFP expression; white arrow indicates a shoot without RFP expression. Observation of the *gl2* knock-out phenotype on a T0 regenerated plant **(C)** and a wild-type seedling after water spray **(D)**. Observations of pollen grains collected from a T0 plant **(E,F)** and a wild-type B104 control **(G,H)**. Images of **(E,G)**, bright field; **(F,H)**, RFP field overlay.

Due to high auto-fluorescence from green chlorophyll, RFP expression on the shoot was screened before transferring to the rooting medium and being incubated under the light. RFP expression on pale-yellow developing shoots was observed (Figures 6A,B). Interestingly, not all transgenic plantlets showed RFP expression on the developing shoots. Regardless of the RFP expression, all shoots that were bigger than 2–3 cm with fully developed leaves were transferred to a rooting medium containing 2 mg/L bialaphos (28–32 days post-infection). In the rooting medium, the bialaphos concentration was reduced to 2 mg/L for root development (13158B2, Table 2). After 7 days on the rooting medium (35–39 days post-infection), roots started emerging from the base of the green shoots. Regenerated plantlets with 4–5 cm long mature roots (Figure 4B) were transferred to the growth chamber for acclimatization (48–55 days post-infection). Well-established plants were transferred to the greenhouse for further growth.

After 2–3 months in the greenhouse, transgenic plants started to produce pollen. RFP expression was checked on the collected pollen grains under the fluorescent microscope. Pollen from the T0 plants showed RFP expression indicating the presence of the T-DNA in the gametes (Figures 6E–H). Using this protocol, established transgenic T0 plants were obtained about 50 days after infection (Table 4). It usually takes additional 3–4 months in the greenhouse to produce mature T1 seeds.

### Targeted Mutagenesis in T0

**FIGURE 7:**
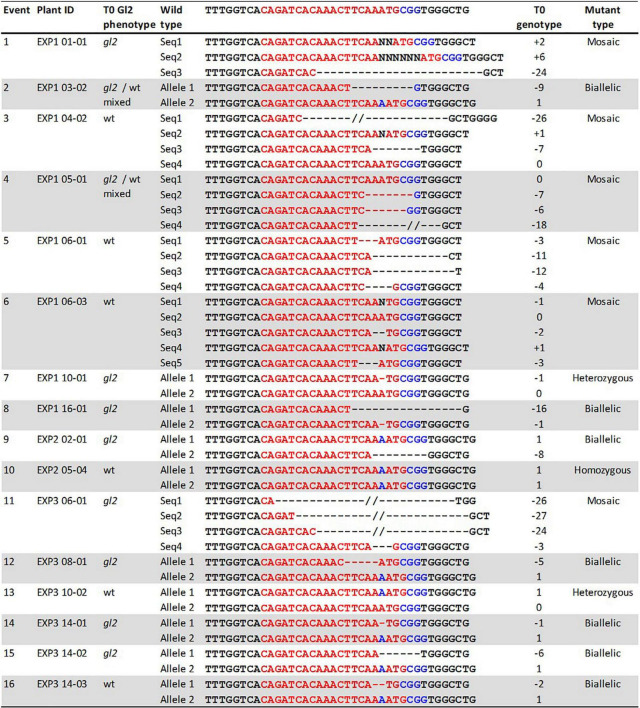
Phenotypes and genotypes of sixteen T0 mutant events. Plant ID shows experiment ID, embryo ID, and plantlet ID (EXP-embryo-plantlet). Three *gl2* phenotypes: *gl2, glossy* mutant phenotype; *gl2*/wt mixed, both *glossy* mutant and wild type phenotype on the same leaf; wt, wild-type phenotype. Red letters, target sequences in *Gl2* exon2; Blue letters, PAM sequences; Black letter, insertion mutations; dashed lines, deletions. T0 *gl2* phenotyping is considered a preliminary screening assay.

Transformation with the construct pKL2013 carrying a Cas9/sgRNA cassette targeting the *Gl2* gene in maize (McCaw et al., 2021) resulted in the generation of 24 transgenic plants. These plants were characterized by Sanger sequencing to determine the targeted mutagenesis outcomes. The *Gl2* exon2 region was PCR amplified from all *Cas9*-positive transgenic plants and a wild-type control. The amplified PCR products were subjected to direct Sanger sequencing. Because the T0 plant can have mixed mutation types on the targeted gene, direct sequencing of PCR products containing such mutations often results in superimposed chromographs. To interpret these data generated from the direct sequencing, the presence of indel mutations at the target site was analyzed by TIDE (Brinkman et al., 2014) and ICE (Hsiau et al., 2019) software using the sequencing trace files and the default parameters. As shown in Table 5, **16** out of 24 (66.6%) *Cas9*-positive plants carried indel mutations, demonstrating that our improved protocol can be used for targeted mutagenesis applications. Among the mutant plants, seven were biallelic (29.2%), six were mosaic (25%), two were heterozygous (8.3%), and one was homozygous (4.2%) (Table 5). Detected mutations were either 1 bp insertion (A) or short deletions ranging from 1 to 27 bp at the target site (Figure 7). Interestingly, three transgenic plants regenerated from the same embryo were carrying different indel mutations (events 14–16 in Figure 7). All three were biallelic mutants and carried an identical 1 bp insertion on one allele but contained different deletion mutations on the other allele.

**TABLE 5:** Summary of T0 mutant genotypes.*

	Number of plants	% T0 mutant
Homozygous	1	4.2%
Biallelic	7	29.2%
Heterozygous	2	8.3%
Mosaic	6	25.0%
Wild type	8	33.3%
**Total analyzed**	**24**	**100.0%**

*Homozygous, one mutant sequence without wild type allele; Biallelic, two different mutant sequences; Heterozygous, wild type sequence and one mutant sequence; Mosaic, three or more mutant sequences in a single plant.

The indel mutations on both alleles of the *Gl2* gene target region can lead to the loss-of-function *gl2* phenotype, in which the juvenile leaf surface becomes dull and does not repel water droplets (Figures 6C,D). This phenotype can be observed in both T0 regenerated young plants and T1 seedlings of mutant progenies after misting their leaf surfaces. However, it is much easier to ascertain the phenotype in T1 seedlings than in the T0 plants (McCaw et al., 2021). In this work, although we screened the *gl2* mutant phenotype in the T0 plants after the regenerants were acclimated for 10 days (Figure 7), we consider the water spraying assay as preliminary. As shown in Figure 7, eight out of 16 plants (events 1, 7–9, 11, 12, 14, and 15) that displayed the *gl2* phenotype indeed carried indel mutations at the target site. Two plants (events 2 and 4) showed a mixed phenotype. Six plants, three mosaics (events 3, 5, and 6), one homozygous (event 10), one heterozygous (event 13), and one biallelic (event 16) displayed wild-type phenotype. Further analysis on all these plants should be carried out in T1 progenies.

## Discussion

*Agrobacterium*-mediated genetic transformation is an important tool for both fundamental and applied research for maize. The current *Agrobacterium*-mediated transformation protocol for B104 inbred requires about 16–22 weeks to generate rooted transgenic plants with an average TF of 4% (Raji et al., 2018). Here, we described a rapid *Agrobacterium*-mediated transformation protocol for B104 including detailed media compositions as well as the stepwise procedures of producing transformation starting materials, preparing *Agrobacterium* cultures, conducting infection and regeneration experiments, phenotyping and genotyping of transgenic and mutant plants, and generating T1 seeds. This much-simplified protocol requires only about 7–10 weeks to produce rooted transgenic plants with an average TF of 6.4%.

In this study, CRISPR/Cas9 reagents targeting *Gl2* gene were delivered into maize embryos to further demonstrate the utility of this protocol in genome editing applications. The overall frequency by CRISPR/Cas9-induced indel mutations was 66.6% (16/24 T0 plants) demonstrating that this rapid B104 transformation protocol can be effective for targeted mutagenesis. Compared to McCaw et al. (2021), which used pKL2013 for targeted mutagenesis in a different genotype, Fast-flowering mini maize, the overall targeted mutagenesis frequency is slightly lower (66.6 vs. 79.1%; *P* > 0.05, two proportion *z*-test). Interestingly, the combined frequency (33.4%) of biallelic (4.2%) and homozygous (29.2%) mutants observed in this study (Table 5) was much lower compared to the 66.3% combined frequency reported in Table 2 of McCaw et al. (2021). This difference is statistically significant (33.3 vs. 66.3%; *P* < 0.01, two proportion *z*-test). This observation may suggest that the reduced callus propagation period in this protocol might have an impact on the efficacy of the CRISPR/Cas9 reagents. The shorter duration of the *in vitro* tissue culture could reduce the chance of targeted mutagenesis by the CRISPR reagents integrated into the genome of the transformed tissues. Nevertheless, loss-of-function mutants were readily obtained in T0 generation with this protocol. A protocol with 50% reduced turnaround time and simplified procedures are clearly advantageous for most genome-editing applications in B104.

The improved transformation protocol could be partially attributable to the ternary helper plasmid, pKL2299, which carries extra copies of essential *Agrobacterium vir* genes used in this study, and adoption of the media used in QuickCorn method (Hoerster et al., 2020; Masters et al., 2020). Various combinations of phytohormones (auxins and cytokinins) in each medium might promote shoot regeneration at the maturation stage reducing the callus selection and proliferation timeline from 161 to 42 days (Table 4, Steps 4–9). For example, selection media used in the previous B104 protocols (Frame et al., 2006; Raji et al., 2018) do not contain cytokinin, while the maturation medium used in this protocol has three cytokinins: zeatin, thidiazuron, and 6-benzylaminopurine. Another explanation for the reduced tissue culture period in this protocol is that the QuickCorn media used in this work induce direct somatic embryogenesis. In the direct somatic embryogenesis process, somatic embryos can form from the explant without the formation of an intermediate callus phase (Raghavan, 1986; Zhang et al., 2021). It is likely that this improved protocol avoids the conventional callus induction and proliferation steps required by the previous B104 transformation protocol (Raji et al., 2018). The major difference of this improved B104 protocol vs. the QuickCorn method is that no morphogenic genes such as *Bbm* and *Wus2* are used in the transformation vectors.

One of the key factors for successful maize transformation is the production of quality immature embryos as starting materials. Here we described the detailed procedure to produce immature embryos for transformation. We recommend using three or more ears for each transformation experiment to reduce the ear-to-ear variation often observed in maize transformation using immature embryos (Frame et al., 2002). The size of immature embryos is another important factor. It is critical to use ears with the right size of embryos (Ishida et al., 2007; McCaw et al., 2021). For *Agrobacterium*-mediated transformation, the typical embryo size is 1.5–1.8 mm for Hi II (Frame et al., 2002) and 1.8–2.0 mm for B104 (Raji et al., 2018). We observed a much lower transformation frequency when small embryos (<1.8 mm) were used in the second experiment. The transformation frequency of the second experiment (EXP2-CAS, Table 3) was significantly lower (1.8%) than the first and the third infection experiments (9.1 and 8.5%, respectively; *P* < 0.05, two proportion *z*-test). Therefore, immature embryos with 1.8–2.0 mm in size are suitable for B104 transformation using this rapid protocol, consistent with the previous recommendation (Raji et al., 2018).

In this study, we observed a high rate of escapes, that is, only 33.3% (24/72) regenerated shoots were *Cas9*-positive transgenic plants. This might be due to the *bar* gene/bialaphos selection system in the binary vector. This non-selective herbicide acts as a glutamine synthetase inhibitor in plants and causes toxicity by accumulating ammonia in the affected plant cells (Donn and Köcher, 2002). The bialaphos selection in this protocol successfully inhibited plant regeneration from the non-infection controls, however, it was less effective in killing the non-transgenic plants from the infected embryos. This could be due to the shortened callus propagation and selection duration in this protocol or the herbicide bialaphos might not metabolize quickly enough to inhibit the rapidly growing plant tissues. To suppress the non-transgenic plant growth, a higher concentration of bialaphos is recommended for the first maturation medium (13329B3). Alternatively, other fast-acting herbicide resistance genes and selection agents such as *HRA* gene with imazapyr (Lowe et al., 2018; Masters et al., 2020) or antibiotics selection system *NPTII* gene with G418 (Wang et al., 2020) can be adapted to this protocol.

In summary, we presented an improved B104 transformation protocol as an effective and rapid method to generate transgenic and genome-edited maize plants. This protocol is fast, with a short turnaround time of about 50 days, from the day of infection to obtaining rooted transgenic plants. We expect this method can benefit the maize research community, especially academic laboratories to generate transgenic and CRISPR-edited maize plants for both fundamental and applied research.

## Data Availability Statement

The original contributions presented in the study are included in the article, further inquiries can be directed to the corresponding authors.

## Author Contributions

KW, MK, and VV designed the experiments. KL designed and built the constructs and *Agrobacterium* strain. MK and TF performed transformation and regeneration experiments. HC provided corn ears and contributed to the plant care. MK and KL performed phenotyping, genotyping, and data analysis of the plants. KW and VV coordinated the project. MK, KW, KL, TF, and VV prepared the manuscript. All authors contributed to discussion and revision of the manuscript.

## Conflict of Interest

The authors declare that the research was conducted in the absence of any commercial or financial relationships that could be construed as a potential conflict of interest.

## Publisher’s Note

All claims expressed in this article are solely those of the authors and do not necessarily represent those of their affiliated organizations, or those of the publisher, the editors and the reviewers. Any product that may be evaluated in this article, or claim that may be made by its manufacturer, is not guaranteed or endorsed by the publisher.

## Footnotes

1
https://www.ars-grin.gov/
